# Reevaluating Gait Alterations in Catatonia with 3D Optical Marker-less Motion Tracking – Are gait alterations clinically overestimated?

**DOI:** 10.1192/j.eurpsy.2025.454

**Published:** 2025-08-26

**Authors:** D. C. Akkoc Altinok, K. Ohl, S. Volkmer, G. Brandt, S. Fritze, D. Hirjak

**Affiliations:** 1 Central Institute of Mental Health, Medical Faculty Mannheim, University of Heidelberg; 2 German Centre for Mental Health (DZPG), partner site, Mannheim, Germany

## Abstract

**Introduction:**

The 3-dimensional (3D) marker-less motion capturing (MoCap) systems are an emerging technique in movement analysis. Recent studies have shown that such MoCap assessment have great potential for accurate and objective assessment of motor alterations in psychiatric patients. Gait as a fundamental gross motor function is the final motor outputs of a complex network integrating sensorimotor systems, cognitive and affective stimuli. Understanding gait alterations in catatonia patients can provide insights into their sensorimotor dysfunction and help in diagnosis and treatment in future.

**Objectives:**

The primary objective of this study is identifying gait alterations using a 3D optical marker-less MoCap system in catatonia patients (CAT) compared to non-catatonia controls (Non-CAT). We hypothesize that there will be significant reduction in the spatiotemporal parameters of gait, such as speed, stride width, stride length, step length, and cadence as well as increased gait instability in CAT group. Moreover, these alterations will be correlated with general psychomotor slowing.

**Methods:**

In this cross-sectional study, we examined a total of 61 patients with and without catatonia diagnosis (CAT; n=22, Non-CAT; n=39) according to ICD-11. Participants were assessed with the Positive and Negative Syndrome Scale (PANSS), Northoff Catatonia Rating Scale (NCRS), the Brief Psychiatric Rating Scale (BPRS), Global Assessment of Functioning (GAF). We conducted clinical psychomotor examination with the Heidelberger Neurological Soft Sign Scale (NSS), Salpêtrière Retardation Rating Scale (SRRS) and Unified Parkinson Disease Rating Scale (UPDRS). Gait analysis were performed using a marker-less MoCap system with 8 cameras (Qualisys, Goeteborg, Sweden). Participants walked on a marked path (18 m) with a self-selected tempo during the experiment. The spatiotemporal parameters (STP) of gait were obtained automatically through Theia Software.

**Results:**

The two groups did not differ in age, sex, or BMI. The CAT group showed higher scores in PANNS, BPRS, NCRS and GAF (p< 0,05). We found a statistically significant difference in clinical psychomotor parameters in NSS, SAS and UPDRS between the two groups (p <0,05) and more specifically in gait subdomain of each scale. No differences were found in any of STP of gait or gait instability measurements after controlling for covariates (age, sex, BMI and medication).

**Image 1:**

**Figure:**
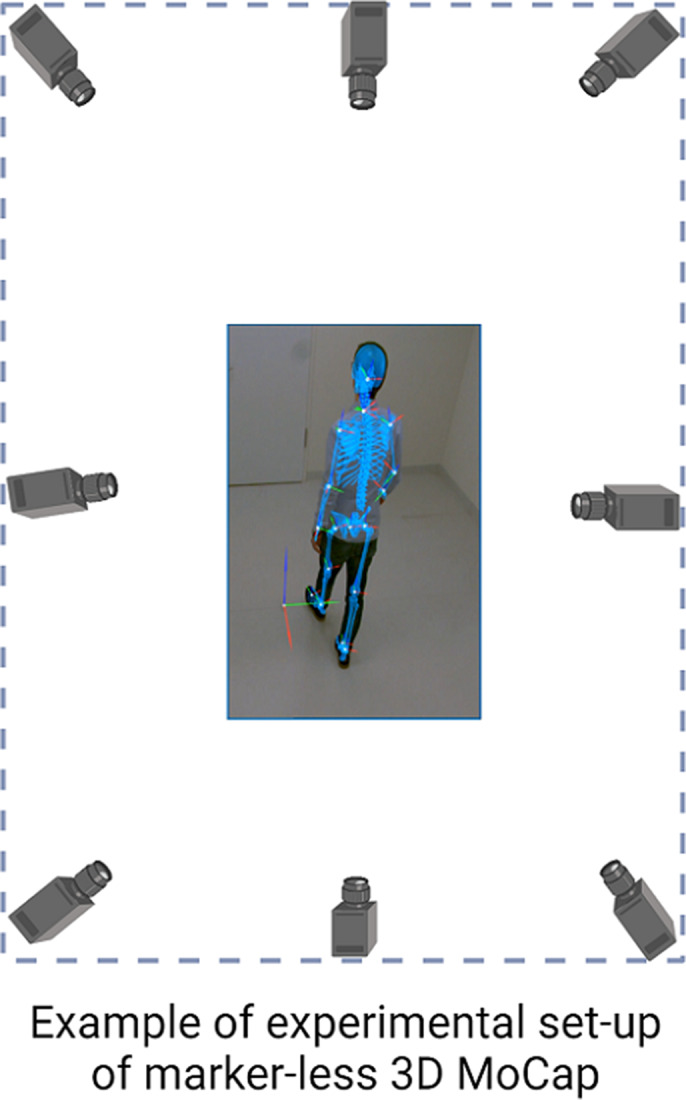

**Conclusions:**

This study exhibit a significant difference between the results of clinical psychomotor assessments and objective gait analysis through marker-less MoCap. This discrepancy suggests a potential overestimation of gait alterations in clinical evaluations. The findings underscore the importance of integrating advanced 3D MoCap technologies into the psychiatric assessment to enhance our understanding of gait alterations in mental disorders.

**Disclosure of Interest:**

None Declared

